# Histopathological analysis of CD163 and myeloperoxidase expression in neutrophil-rich dermatoses^؆^

**DOI:** 10.1016/j.abd.2026.501425

**Published:** 2026-08-17

**Authors:** Danielle Carvalho Quintella, Cristiane Bedran Milito, Tullia Cuzzi

**Affiliations:** Department of Pathology, Faculty of Medicine, Universidade Federal do Rio de Janeiro, Rio de Janeiro, RJ, Brazil

**Keywords:** Immunohistochemistry, Leprosy, Sporotrichosis, Sweet syndrome, Vasculitis, leukocytoclastic, cutaneous

## Abstract

**Background:**

Predominance of myeloperoxidase immunoreactive mononucleated cells in the dermal infiltrate defines histiocytoid Sweet’s syndrome. These cells are believed to represent immature myeloid cells of granulocytic lineage, and the authors suppose that they could be present in other neutrophil-rich inflammatory dermatoses.

**Aim:**

This report aimed to investigate CD163 and/or MPO expression on mononuclear cells in dermatoses with tissue neutrophilia not previously considered.

**Methods:**

A double immunostaining protocol with CD163 and MPO primary antibodies was applied to skin biopsies of 4 groups of neutrophil-rich dermatoses: Sweet’s syndrome, acute neutrophilic vasculitis, type 2 leprosy reaction, and sporotrichosis.

**Results:**

CD163 + MPO− and CD163 + MPO+ macrophages were present in all groups, ranging from less than 10% to more than 50% and from less than 10% to 10%‒50% of positive cells, respectively. CD163 − MPO+ histiocytoid cells were detected in all groups, except in the acute neutrophilic vasculitis group.

**Study limitations:**

The present study was unicentric with a small number of cases, and then loss of a more refined statistical analysis.

**Conclusions:**

Besides the identification of MPO immunoreactive macrophages by a double immunostaining protocol, CD163 − MPO+ histiocytoid cells were found not to be restricted to classic neutrophilic dermatosis, and tissue neutrophilia does not imply the presence of immature myeloid cells.

## Introduction

Mononucleated cells with elongated or reniform nuclei were noted in the dermal cell infiltrate of Sweet’s syndrome (SS). Initially, considered in the context of coexisting leukemia cutis, they were later interpreted as corresponding to immature myeloid cells of granulocytic lineage.^1^, ^2^ Thus, a histopathological variant of the disease was proposed, histiocytoid SS (HSS), defined by a dermal infiltrate mostly composed of myeloperoxidase (MPO) immunoreactive mononucleated cells.^3^

To define the lineage of histiocytoid cells, Alegría-Landa et al. studied 33 cases of HSS and demonstrated, through double immunohistochemical staining, that most cells of the predominantly mononuclear dermal infiltrate belonged to the granulocytic lineage.^4^ Furthermore, the coexpression of CD163 and MPO was found only in 6 cases, corresponding to less than 10% of the cells. Contrary to others, a predominance of macrophages was not reported.^5^, ^6^, ^7^ Thus, that series of cases corroborated the interpretation that MPO+ mononucleated cells would correspond to immature precursors of neutrophils, predominant in HSS.^4^

However, the recurrent finding of similar cells, but morphologically and immunophenotypically consistent with macrophages, in SS cases called attention.^8^ Thus, many authors questioned whether mononucleated cells with reniform nuclei, called histiocytoid, would be precursors of neutrophils or MPO+ macrophages.^5^, ^6^

MPO+ mononucleated cells were described in pyoderma gangrenosum, but additional studies that focused on the participation of those cells in other neutrophil-rich inflammatory dermatoses are not available.^9^ This report aimed to verify whether their presence could be extended to additional processes with tissue neutrophilia and to investigate the expression of CD163 and/or MPO on mononuclear cells present in dermatoses not previously reported.

## Materials and methods

This cross-sectional observational study was approved by the local Research Ethics Committee (record #3.115.862).

Skin biopsies, referred to as cases, performed between 01/01/2000 and 31/12/2018, were included and distributed into four different groups according to the histopathological diagnosis consistent with clinical suspicion: SS, acute neutrophilic vasculitis (ANV), type 2 leprosy reaction (T2LR), and sporotrichosis (S). A positive culture for *Sporothrix* sp. was obtained for all the S group cases.

Cases in which the histopathological diagnosis was not confirmed after clinical, laboratory, and/or evolutive data were excluded.

All cases were reviewed for histopathological aspects (H&E staining), including the evaluation of cell types constituting the dermal infiltrate.

Double immunostaining was carried out with a monoclonal mouse CD163 (Cell Marque, #163 M-14, 1:200 dilution) and polyclonal rabbit MPO (Dako, #A-0398, 1:7000 dilution) as primary antibodies. A polymer detection system was applied accordingly (Nicherei Bioscience, #414132F; Nicherei Bioscience, #414252F). Positive CD163 and MPO reactions were revealed by brown DAB (Leica, #7162) and permanent red (Scy Tek, #PRD-61), respectively. Positive controls for CD163 (tonsil) and MPO (bone marrow) were used.

To evaluate the relative participation of immunophenotypically distinct cells among positive immunostained cells was performed the analysis of the entire lesional dermis at 40× magnification and a semiquantitative analysis was applied for quantification using the following score: 0 if no positive cells were identified, + if less than 10%, ++ if 10% to 50% and +++ if more than 50% positive cells were identified.

The data obtained were imported into IBM-SPSS Statistics software, version 26 (IBM Corp.), and the R program.^10^ The chi-square test was applied for comparative analysis of the groups, and a p-value of 5% was considered.^11^

## Results

Seventy-nine cases studied were distributed into 4 groups: the SS group (*n* = 25) with an evolution time between 3- and 21-days, the ANV group (*n* = 15) with an evolution time between a few hours and 1-year, the T2LR group (*n* = 19), and the S group (*n* = 20). Complete available data about the lesion’s duration, resolution, and treatment were presented as supplementary material (Supplementary Tables 1–4).

A review of H&E-stained slides of the SS group showed confluent inflammatory infiltrate in the upper dermis or a diffuse dermal distribution of the cells in 14/25 cases. In the remaining cases, dermal infiltrate was perivascular or perivascular and interstitial only. Focal fibrinoid necrosis of the vessel wall was observed in 6/25 of the cases. In addition, papillary dermal edema (17/25), leukocytoclasis (25/25), hemorrhage (24/25), and endothelial swelling (10/25) were observed. Histiocytoid mononuclear cells predominated in 7 cases.

In the ANV group, neutrophils were present around vessels with infiltration of their wall, associated with fibrinoid necrosis (8/15) and/or endothelial swelling (2/15). In addition, papillary dermal edema (3/15), hemorrhage (15/15), and leukocytoclasis (12/15) were observed. Mononuclear inflammatory cells were noted in 12/15 cases.

The T2LR group was characterized by a mononuclear dermal-hypodermal cell infiltrate mostly consisting of foamy cells (Virchow cells), accompanied by neutrophils. Focal fibrinoid necrosis of the vessel wall (3/19), papillary dermal edema (9/19), hemorrhage (14/19), leukocytoclasis (8/19), and endothelial swelling (9/19) were also present. In all cases, intact or fragmented acid-fast bacilli were seen (Wade stain).

Sporotrichosis lesions were mainly represented by diffuse granulomatous dermatitis together with a dense mixed inflammatory infiltrate. Suppurative granuloma (15/19 cases), pseudoepitheliomatous hyperplasia (7/20), and/or ulceration (9/20) were additional findings. Fungal spores were seen in 12/20 cases (PAS and/or Grocott's methenamine silver stains).

**Fig. 1:**
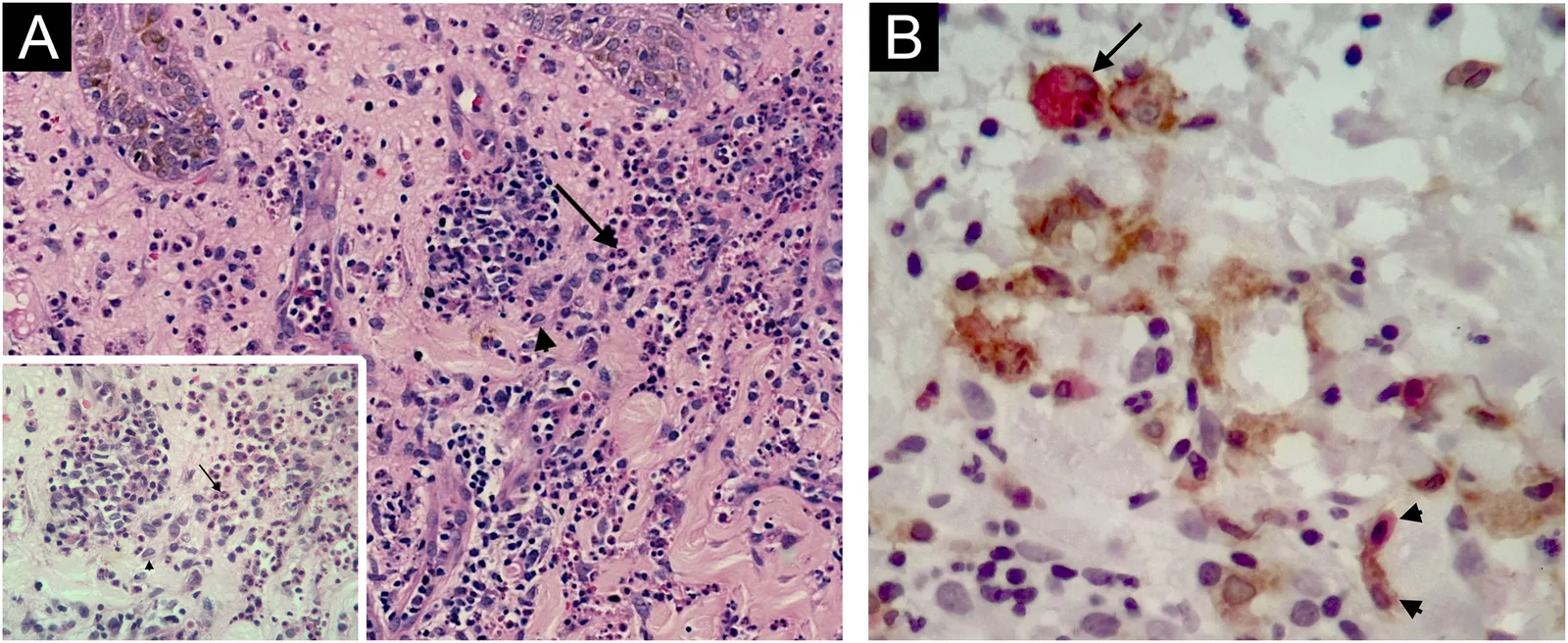
Sweet’s syndrome. Case 10. (A) Perivascular and interstitial inflammatory infiltrate of neutrophils (arrow) and histiocytoid cells (arrow-head). (B) CD163 + MPO+ macrophages (arrow), CD163 + MPO− and CD163 − MPO+ histiocytoid cells (arrow heads). Hematoxylin & eosin, 200×; Hematoxylin & eosin detail and double immunostaining, 400 × .

Double staining with CD163 and MPO primary antibodies in the SS group revealed CD163 + MPO− macrophages in all cases, making up more than 50% of cells in 92% (23/25) of cases. CD163 − MPO+ histiocytoid cells were seen in 28% (7/25) of the cases, representing less than 10% of the cells in 57.1% (4/7) of cases. CD163 + MPO+ macrophages were observed in 88% (22/25) of the cases, representing less than 10% of the cells in 81.8% (18/22) of the cases (Fig. 1).

**Fig. 2:**
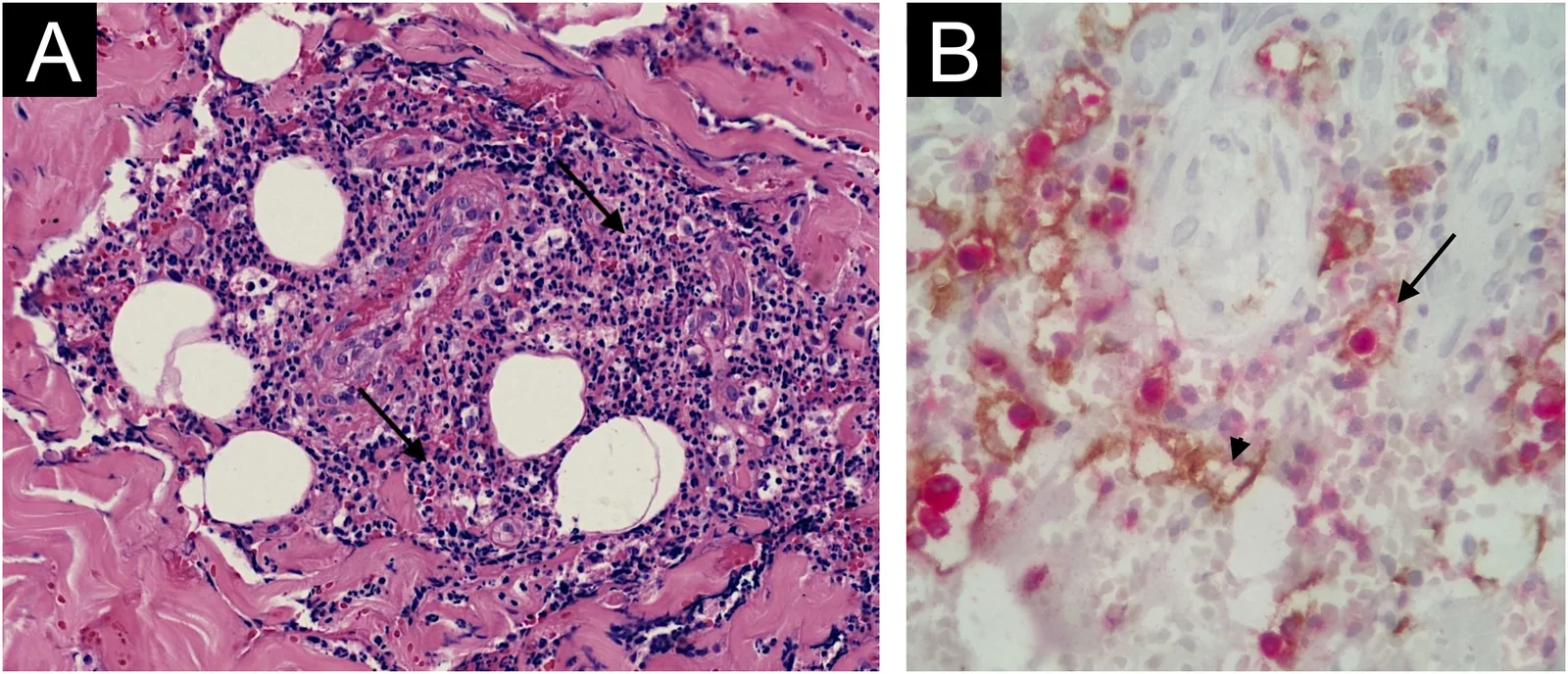
Acute neutrophilic vasculitis. Case 10. (A) Neutrophilic perivascular infiltrate (arrows). (B) CD163 + MPO− (arrow-head) and CD163 + MPO+ macrophages (arrow). Hematoxylin & eosin, 200×; Double immunostaining, 400×.

In the ANV group, CD163 + MPO− macrophages were observed in all cases. CD163 − MPO+ histiocytoid cells were not observed. CD163 + MPO+ macrophages were observed in 60% (9/15) of the cases, representing less than 10% of the cells in 66.7% (6/9) of the cases (Fig. 2).

**Fig. 3:**
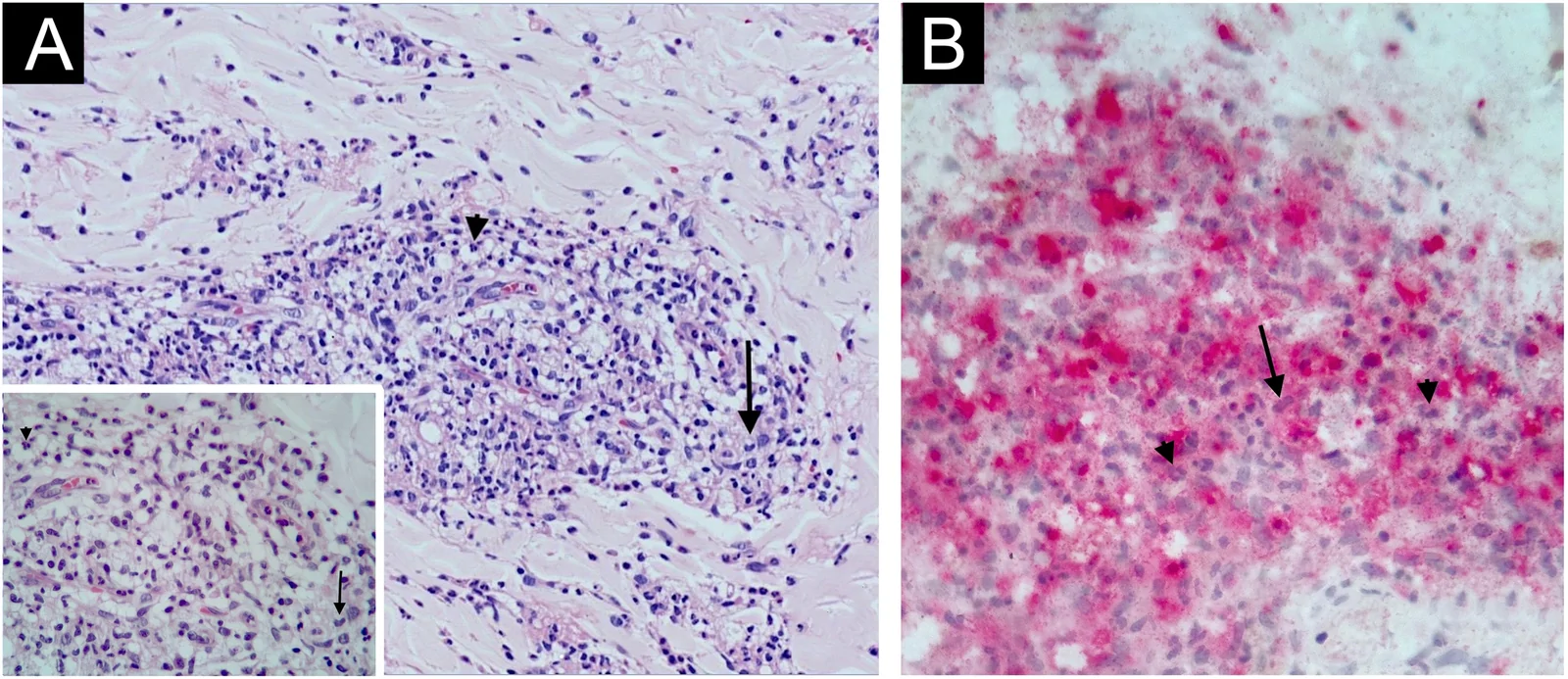
Type 2 leprosy reaction. Case 2: (A) Numerous histiocytoid cells (arrow) and neutrophils (arrow-head) around vessels. (B) CD163 − MPO+ polymorphonuclear (arrow heads) and histiocytoid cells (arrow). Hematoxylin & eosin, 200×; Hematoxylin & eosin detail and double immunostaining, 400×.

CD163+MPO− macrophages were observed in all cases of the T2LR group, composing more than 50% of the cells in 68.4% (13/19) of cases. CD163 − MPO+ histiocytoid cells were seen in 78.9% (15/19) of the cases, representing 10%‒50% of the cells in 66.7% (10/15) of cases. CD163 + MPO+ macrophages were observed in 84.2% (16/19) of the cases, composing less than 50% of the cells in all cases (Fig. 3).

**Fig. 4:**
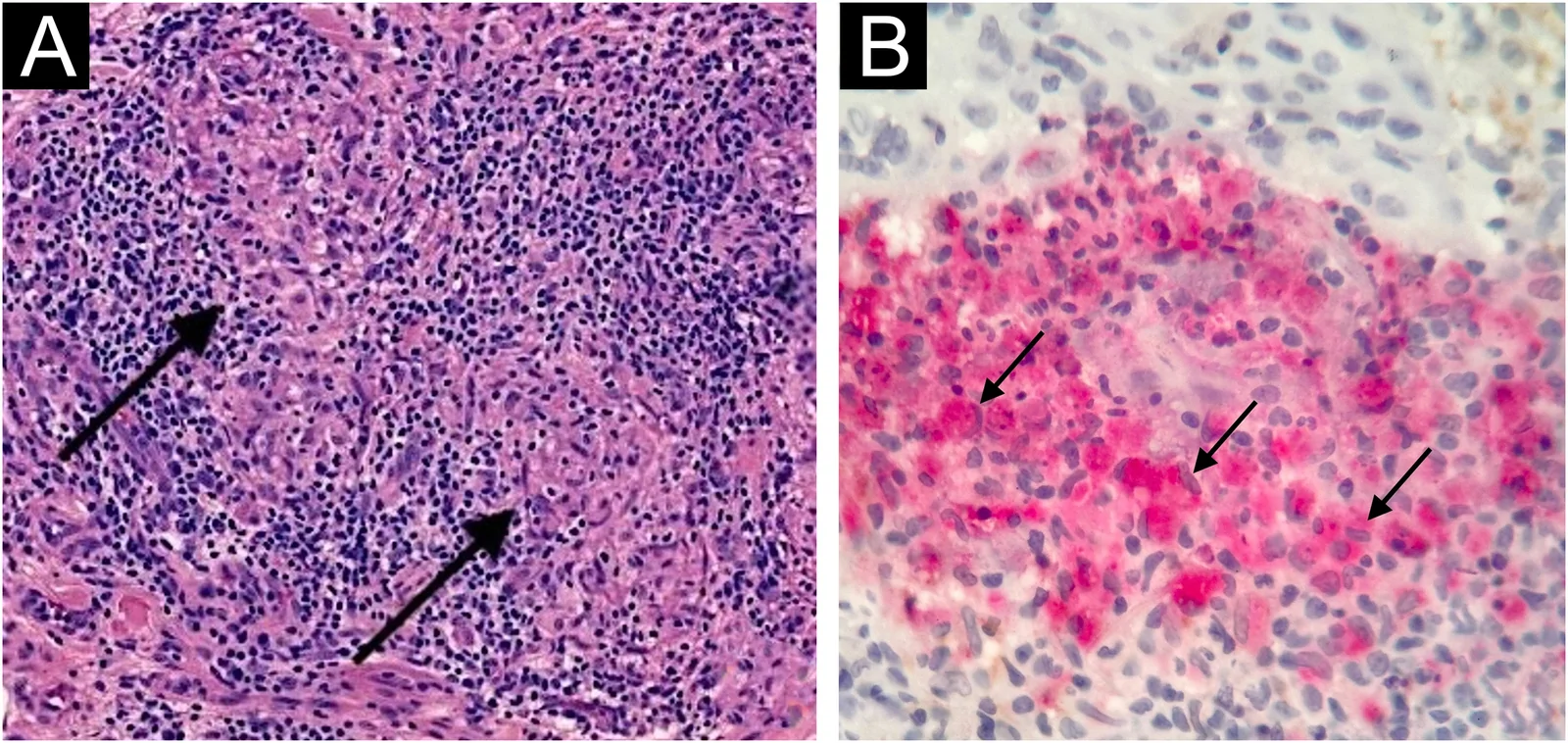
Sporotrichosis. Case 1: (A) Histiocytoid grouped cells (arrows). (B) CD163 − MPO+ histiocytoid cells. Hematoxylin & eosin, 200×; Double immunostaining, 400×.

CD163+MPO− macrophages were also observed in all cases of the S group, representing more than 50% of cells in 80% (16/20) of cases. CD163 − MPO+ histiocytoid cells were seen in 30% (6/20) of the cases, representing less than 10% of the cells (Fig. 4). CD163 + MPO+ macrophages were observed in 35% (7/20) of the cases, representing less than 10% of the cells. Detailed immunohistochemical findings are presented in Table 1.

**Table 1:** Results of the immunohistochemical study in the four groups studied.

Morphology and immunohistochemical profile		SS (*n* = 25)	ANV (*n* = 15)	T2LR (*n* = 19)	S (*n* = 20)	p-value
CD163 + MPO− Macrophages	0	0 (0%)	0 (0%)	0 (0%)	0 (0%)	0.007
	+	2 (8%)	2 (13.3%)	0 (0%)	0 (0%)	
	++	13 (52%)	10 (66.7%)	6 (31.6%)	4 (20%)	
	+++	10 (40%)	3 (20%)	13 (68.4%)	16 (80%)	
CD163 − MPO+ Histiocytoid cells	0	18 (72%)	15 (100%)	4 (21.1%)	14 (70%)	<0.001
	+	4 (16%)	0 (0%)	5 (26.3%)	6 (30%)	
	++	3 (12%)	0 (0%)	10 (52.6%)	0 (0%)	
	+++	0 (0%)	0 (0%)	0 (0%)	0 (0%)	
CD163 − MPO+ Polymorphonuclear cells	0	0 (0%)	2 (13.3%)	1 (5.3%)	4 (20%)	<0.001
	+	1 (4%)	0 (0%)	3 (15.8%)	9 (45%)	
	++	13 (52%)	5 (33.3%)	15 (78.9%)	6 (30%)	
	+++	11 (44%)	8 (53.4%)	0 (0%)	1 (5%)	
CD163 + MPO+ Macrophages	0	3 (12%)	6 (40%)	3 (15.8%)	13 (65%)	<0.001
	+	18 (72%)	6 (40%)	8 (42.1%)	7 (35%)	
	++	4 (16%)	3 (20%)	8 (42.1%)	0 (0%)	
	+++	0 (0%)	0 (0%)	0 (0%)	0 (0%)	

SS, Sweet’s Syndrome; ANV, Acute Neutrophilic Vasculitis; T2LR, Type 2 Leprosy Reaction; S, Sporotrichosis; MPO, Myeloperoxidase; 0, No positive cells identified; +, Less than 10% positive cells; ++, 10%‒50% positive cells; +++, More than 50% positive cells.

## Discussion

Histological findings resulting from the review of the H&E-stained sections, along with clinical and/or laboratory compatibility, validate the location of the cases in the 4 proposed groups. Clinically distinct diseases have been arranged under the descriptive designation neutrophilic dermatoses based on the predominance of neutrophils in the dermal inflammatory infiltrate and the absence of vasculitis.^12^ Acute febrile neutrophilic dermatosis, also called SS, deserves special mention since it is more frequently observed and on which initial investigations were made to better understand the events that could justify the absence of predominance of neutrophils in favor of the presence of mononucleated cells with a histiocytoid morphology. Such cells, considered to be immature myeloid cells of granulocytic lineage, justified the HSS histological variant of the disease when composing most of the dermal infiltrate.^1^, ^2^, ^3^ However, the investigation of those histiocytoid cells was not expanded to other dermatoses with tissue neutrophilia. In addition, their histogenesis seems not to be completely established since others argued for MPO-immunoreactive macrophage.^5^, ^6^

CD163 − MPO+ histiocytoid cells are not restricted to entities histologically represented by a predominant neutrophilic inflammatory infiltrate, since they were detected in cases of the T2LR and S groups. Dermatoses with a more acute clinical course and histologically accompanied by dominant neutrophils could generate an overloaded demand on the bone marrow, as proposed for HSS.^3^ On the other hand, CD163 − MPO+ histiocytoid cells were not seen in the ANV group, meaning that these cells don’t seem to play a role in ANV, and infiltrates mostly composed of neutrophils do not ensure the presence of their precursors in tissue. Particularly interesting is the finding of numerous CD163 − MPO+ histiocytoid cells in the T2LR group, which may correlate with ongoing systemic and acute events in chronic disease otherwise histologically characterized by a macrophage infiltrate. Accordingly, in the S group, which represents another chronic disease with macrophage-rich infiltrate but lacks acute intervening phenomena, a small number of CD163 − MPO+ histiocytoid cells were seen. These distinct findings possibly reflect qualitative and quantitative differences in the mechanisms that interfere with the recruitment and release of immature cells by the bone marrow, such as those related to the granulocyte colony-stimulating factor.^13^, ^14^, ^15^, ^16^ Details about the physiopathology of the diseases included in this study are beyond our scope. Regarding the SS group, CD163 − MPO+ histiocytoid cells were not found in sufficient numbers to allow the diagnosis of HSS in any of the cases, despite an observed predominance of mononuclear cells on H&E-stained sections in 7 of them.

CD163 + MPO− macrophages were present in all cases included in this study in a more numerous cell proportion, a finding that matches their importance even in the initial moments of the inflammatory process. Accordingly, statistical significance (p-value) was not achieved between the groups.

**Fig. 5:**
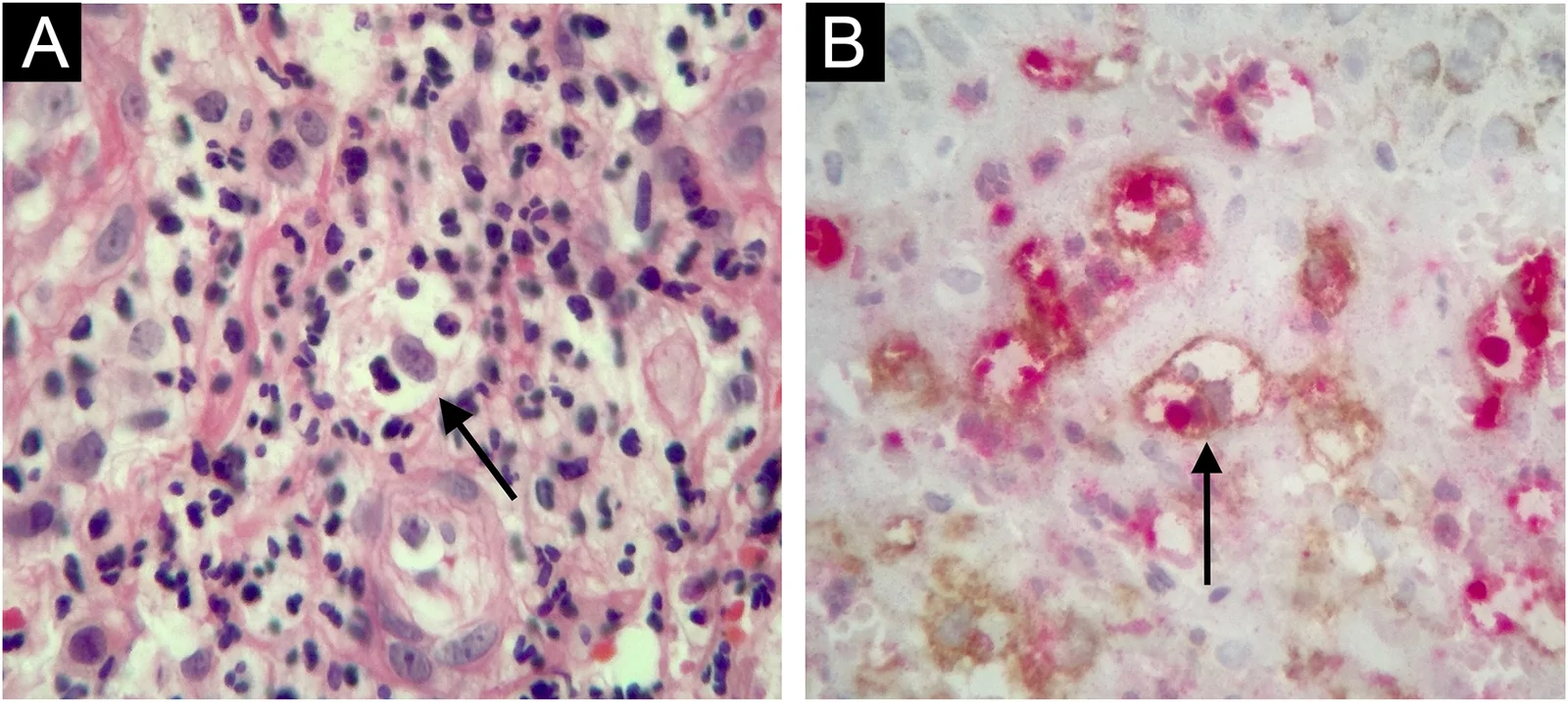
Acute neutrophilic vasculitis. Case 10: (A and B) Macrophage containing neutrophils or their remains (arrow). Hematoxylin & eosin and double immunostaining, 400×.

The immunophenotype CD163 + MPO+, consistent with macrophages containing degenerated neutrophils or their remains, was found in at least some cases of all groups (Fig. 5). Those CD163 + MPO+ cells were observed in the ANV group and most of the cases of SS, in contrast to what was described by Alegría-Landa et al., the reference most similar to the present study.^4^

Macrophages can mimic neutrophils, and the morphological differential diagnosis between small macrophages and myeloid precursors may be challenging, which justifies the immunohistochemical approach to differentiate those cell types.^8^ In fact, regarding the SS group, CD163 − MPO+ histiocytoid cells were not found in sufficient numbers to allow the diagnosis of HSS in any of the cases, despite an observed predominance of mononuclear cells on H&E-stained sections in 7 of them. Additionally, CD163 + MPO+ macrophages could be misinterpreted as myeloid precursors in a more conventional immunohistochemical protocol using only MPO as the primary antibody. Our study stresses the importance of performing a double immunohistochemical stain to better characterize histiocytoid cells and recognize myeloid precursor cells whose presence in inflammatory dermatosis with tissue neutrophilia remains to be further investigated. For practical diagnostic purposes, given no specificity of the CD163 − MPO+ histiocytoid cells finding, appointment of HSS should follow original description together with clinical support. Clinical diagnosis of SS may be challenging with consideration of other diseases. Predominance of mononuclear cells on H&E-stained slides of SSH would technically disqualify the reaction pattern as a neutrophilic dermatosis. Altogether, opens the scenario for a diagnostic impression of other possible non-related dermatoses that may be clinically considered.

Limitations of the present study concern its being unicentric with a small number of cases and the loss of a more refined statistical analysis.

## Conclusion

CD163 − MPO+ histiocytoid cells, thought to correspond to immature myeloid cells of granulocytic lineage, were not restricted to classically designated neutrophilic dermatosis and may be present in other inflammatory diseases with tissue neutrophilia. In addition, tissue neutrophilia did not seem to imply the presence of immature myeloid cells. CD163 + MPO− macrophages outnumbered CD163 − MPO+ histiocytoid cells when both immunophenotypically distinct cells were present. Double immunostaining with CD163 and MPO primary antibodies contributed to a more precise identification of cells to be considered immature myeloid cells of granulocytic lineage versus macrophages participating in the early events of the inflammatory tissue response, with practical differential diagnostic implications.

## Authors' contributions

Danielle Carvalho Quintella: Did study conception and planning; did critical literature review; data collection, analysis, and interpretation; statistical analysis, prepared and wrote the manuscript; did manuscript critical review, and approved the final version.

Cristiane Bedran Milito: Did study conception and planning; data collection, analysis, and interpretation; prepared and wrote the manuscript; did manuscript critical review, approved the final version and effectively participated in research orientation.

Tullia Cuzzi: Did study conception and planning; did critical literature review, data collection, analysis and interpretation; statistical analysis, prepared and wrote the manuscript; did manuscript critical review, approved the final version, and effectively participated in research orientation.

## Financial support

None declared.

## Research data availability

The entire dataset supporting the results of this study was published in this article.

## Conflicts of interest

None declared.
