# Supplementary material for: Histopathological analysis of CD163 and myeloperoxidase expression in neutrophil-rich dermatoses
Source: An Bras Dermatol. 2026 Aug 17;101(5):501425. doi: 10.1016/j.abd.2026.501425 (PMC13507747; doi:10.1016/j.abd.2026.501425)
Supplement: Supplementary file 1 [file mmc1.docx]

ABD-D-25-00176_Supplementary Material

**Supplementary Table 1** Lesion’s duration, time until resolution and treatment in the Sweet syndrome group.

| **Sample** | **Lesion’s duration (days)** | **Treatment** | **Resolution (days)** |
| --- | --- | --- | --- |
| 1 | NI | Tetraciclin | 7 |
| 2 | 7 | Topic corticosteroid | NI |
| 3 | 20 | NI | NI |
| 4 | 7 | NI | NI |
| 5 | 4 | NI | NI |
| 6 | 10 | NI | NI |
| 7 | NI | NI | NI |
| 8 | NI | Oral corticosteroid | 30 |
| 9 | 10 | NI | NI |
| 10 | 15 | NI | NI |
| 11 | 3 | Topic corticosteroid | 30 |
| 12 | 5 | NI | NI |
| 13 | NI | Oral corticosteroid | 40 |
| 14 | NI | Oral corticosteroid | 40 |
| 15 | 14 | Corticosteroid | NI |
| 16 | 5 | Oral corticosteroid | 60 |
| 17 | 5 | Oral corticosteroid | 60 |
| 18 | 4 | Corticosteroid | 60 |
| 19 | 14 | None | NI |
| 20 | NI | Corticosteroid | 7 |
| 21 | 10 | Corticosteroid | NI |
| 22 | 7 | Corticosteroid | NI |
| 23 | 21 | Corticosteroid | NI |
| 24 | 14 | Corticosteroid | 30 |
| 25 | 7 | NI | NI |
| 26 | NI | None | 30 |

NI, Not Informed.

**Supplementary Table 2** Lesion’s duration, time until resolution and treatment in the acute neutrophilic vasculitis group.

| **Sample** | **Lesion’s duration (days)** | **Treatment** | **Resolution (days)** |
| --- | --- | --- | --- |
| 1 | 42 | NI | 30 |
| 2 | NI | Corticosteroid | 60 |
| 3 | 5 | NI | NI |
| 4 | 365 | Corticosteroid | NI |
| 5 | NI | NI | NI |
| 6 | 60 | Corticosteroid, mycophenolate | NI |
| 7 | NI | NI | NI |
| 8 | NI | Mupirocin, moisturizer | 60 |
| 9 | 30 | Corticosteroid, mycophenolate, cyclophosphamide | NI |
| 10 | 6 | NI | NI |
| 11 | NI | Corticosteroid, cyclophosphamide | NI |
| 12 | 4 | Corticosteroid | NI |
| 13 | NI | NI | NI |
| 14 | <1 | None | 60 |
| 15 | NI | Corticosteroid | 1 |

NI, Not Informed.

**Supplementary Table 3** Lesion’s duration, time until resolution, and treatment in the type 2 leprosy reaction group.

| **Sample** | **Lesion’s duration (days)** | **Treatment** | **Resolution (days)** |
| --- | --- | --- | --- |
| 1 | 7 | NI | NI |
| 2 | NI | NI | NI |
| 3 | NI | Corticosteroid | NI |
| 4 | NI | Corticosteroid | NI |
| 5 | NI | Corticosteroid | NI |
| 6 | NI | NI | NI |
| 7 | NI | NI | NI |
| 8 | 14 | Corticosteroid | NI |
| 9 | 21 | NI | NI |
| 10 | NI | Corticosteroid | NI |
| 11 | NI | Corticosteroid, thalidomide | NI |
| 12 | 3 | NI | NI |
| 13 | NI | NI | NI |
| 14 | NI | NI | NI |
| 15 | NI | NI | NI |
| 16 | 2 | Thalidomide | NI |
| 17 | NI | NI | NI |
| 18 | NI | NI | NI |
| 19 | 6 | NI | NI |

NI, Not Informed.

**Supplementary Table 4** Lesion’s duration, time until resolution, and treatment in the sporotrichosis group.

| **Sample** | **Lesion’s duration (days)** | **Treatment** | **Resolution (days)** |
| --- | --- | --- | --- |
| 1 | 90 | Itraconazole | NI |
| 2 | 90 | NI | NI |
| 3 | NI | NI | NI |
| 4 | NI | NI | NI |
| 5 | 90 | Itraconazole | 730 |
| 6 | 46 | NI | NI |
| 7 | 730 | Itraconazole | 60 |
| 8 | 90 | Itraconazole | 120 |
| 9 | 30 | Itraconazole | 60 |
| 10 | 30 | NI | NI |
| 11 | 30 | Heat | NI |
| 12 | 40 | NI | NI |
| 13 | 21 | NI | NI |
| 14 | 120 | NI | NI |
| 15 | 60 | NI | NI |
| 16 | NI | NI | NI |
| 17 | 30 | Itraconazole | 120 |
| 18 | 30 | Itraconazole | NI |
| 19 | 30 | NI | NI |
| 20 | 30 | NI | NI |

NI, Not Informed.
